# 

**Published:** 1895-02

**Authors:** 


MESSRS. SCHULZE-BERGE & KOECHL, 79 Murray street, New York, announce that they are sole agents for the United States for the diphtheria antitoxin-Behring. In order to protect physician and patient they are willing, although contrary to their custom, to sell direct to physicians and retail pharmacists, who are unable to obtain promptly the remedy from their dealers, at the following prices accompanied with cash :

No. 1, green label, 600 antitoxin units, $1.90 ; No. 2, white label, 1,000 antitoxin units, $3.50, and No. 3 red label, 1,500 antitoxin units, $5.25.

---

THE *Atlantic Medical Weekly* is the name of a journal that made its appearance Saturday, January 5, 1895. It is published in Providence, R. I., and is the successor of the *R. I. Medical Science Monthly.* It is edited by Dr. J. Frederick Haller, editor-in-chief ; Dr. Alexander B. Briggs, associate editor, and Edward F. Gamwell, managing editor. Its first numbers present an excellent appearance.

---

MESSRS. JOHNSON & JOHNSON, of New York, have published a very interesting brochure entitled Kola, which gives a history of the plant and its fruit, including its bibliography, which latter occupies seven pages of the pamphlet. A review of this literature is then presented, accompanied by illustrations.

This monograph will be furnished on application to the publishers, Messrs. Johnson & Johnson, 92 William street.

---

THE American Public Health Association, at its last meeting, decided to issue a quarterly journal in place of an annual volume of transactions. The first number appeared in January, 1895, and is well edited and handsomely printed.

Dr. Irving A. Watson, secretary of the association, is the editor, and it is published by the association at Concord, N. H.

---

DR. GEORGE M. GOULD, of 119 South Seventeenth street, Philadelphia, is making a collective investigation as to the influence of albinism upon the eye. He requests all physicians who have had albino patients to address him for a printed sheet of questions.

As it is purely in the interests of science that such work is being done, we hope that all who can will aid the doctor. Any person who can send him the name and address of an Albino will materially help in this good work.

---

THE F. A. Davis's Company, medical publishers, Philadelphia, announces that it will start the new year by issuing, early in February, a companion book to Dr. R. von Krafft-Ebing's famous treatise, Psychopathia Sexualis, entitled Suggestive Therapeutics in Psychopathia Sexualis, it being a translation of the original by Dr. A. Schrenck-Notzing, of Munich, collaborator with Krafft-Ebing. This book will contain about 325 pages and be sold by subscription only, at $2.50 per volume, in cloth. It will be of the greatest importance as an authoritative work on suggestion as a therapeutic agent in the hands of the intelligent practitioner.

---

ALBERT LYNCH, the famous French artist, who received the highest Salon prize for his panel of Spring, has been engaged by *The Ladies' Home Journal* to draw a series of designs for the cover of that magazine, which, as the reading public knows, changes its cover design each month. Lynch is, perhaps, one of the best-paid artists in France, and these covers will cost *The Ladies' Home Journal* nearly $1,000 apiece. But this only demonstrates the enormous expense to which magazines are put in the production of their numbers.—*R. H. Stoddard, in New York Mail and Express.*

---

E. B. TREAT, publisher, New York, has in press for early publication the 1895 International Medical Annual, being the thirteenth yearly issue of this eminently useful work. Since the first issue of this one volume reference work, each year has witnessed marked improvements; and the prospectus of the forthcoming volume gives promise that it will surpass any of its predecessors. It will be the conjoint authorship of thirty-eight distinguished contributors and specialists from America, England and the Continent. It will contain the progress of medical science in all parts of the world, together with a large number of original articles and reviews by authors on subjects with which their scientific reputation is identified. In short, the design of the book is to bring the practitioner into direct communication with those who are advancing the science of medicine, so he may be furnished with all that is worthy of preservation, as reliable aids in his daily work. Illustrations in black and colors will be freely used in elucidating the text. A most useful investment for the medical practitioner. The price remains the same as heretofore, $2.75.

———

MESSRS. WILLIAM WOOD & CO., medical publishers, New York, announce in preparation for immediate publication a work entitled Twentieth Century Practice, which will be an international encyclopedia of modern medical science written by leading authorities in America and Europe. The editor, Dr. Thomas L. Stedman, spent the greater part of 1893 in visiting personally the European contributors to this work. It will be sold by subscription only, for the entire series of twenty volumes.

Prices at which the Twentieth Century Practice will be sold: In extra muslin binding, $5.00; in leather, $6.00; in half-morocco, $7.50; payable invariably on the delivery of the volumes, at, as nearly as possible, regular intervals of three months.

By the plan of publication proposed, this great undertaking is to be completed by the close of the year 1899. So that those who have been so fortunate as to begin their subscription at once, will, upon the most easy terms, find themselves the owners of what it is expected will be the most modern, most practical, most useful and most authoritative work on the practice of medicine at the beginning of the twentieth century.

———

THE *Medical and Surgical Reporter*, of Philadelphia, ends the forty-second year of its consecutive publication with its issue of December 29, 1894. The chief editorial in this number is devoted to a review of the advances made in medicine and surgery during 1894. In speaking of Medical Literature, the editors say:

"Of the making of books there is no end," thanks to the prevision of generous publishers, and the literary crop of the year contains some wheat among the tares. A few works have been issued in special lines whose value justifies their existence. For pronounced excellence, general utility and widest application, Dr. Gould's new medical dictionary deserves specific mention among the publications of 1894.

———

THE *Woman's Medical Journal*, of Toledo, O., has presented us with a handsomely illuminated calendar for the year 1895, for which we beg the editors to accept our best thanks.

